# Identification and analysis of diverse programmed cell death patterns in idiopathic pulmonary fibrosis using microarray-based transcriptome profiling and single-nucleus RNA sequencing

**DOI:** 10.3389/fmed.2025.1534903

**Published:** 2025-06-18

**Authors:** Jiazheng Sun, Yulan Zeng

**Affiliations:** Department of Respiration, Liyuan Hospital, Tongji Medical College, Huazhong University of Science and Technology, Wuhan, China

**Keywords:** idiopathic pulmonary fibrosis, programmed cell death, prognostic signature, diagnostic signature, microenvironment

## Abstract

**Background:**

Idiopathic pulmonary fibrosis (IPF) is a chronic, progressive pulmonary disorder marked by the gradual substitution of lung tissue with fibrotic tissue, resulting in respiratory failure. While the precise etiology of IPF remains unclear, an increasing number of studies have indicated that programmed cell death (PCD) significantly contributes to the onset and advancement of IPF. PCD is implicated not only in the impairment of alveolar epithelial cells during fibrosis but also in the alterations of immune cells inside the fibrotic milieu. Investigating the PCD patterns offers a novel approach to the early diagnosis and prognostic evaluation of IPF.

**Methods:**

The study utilized microarray-based transcriptome profiling and single-nucleus RNA sequencing to identify and analyze diverse PCD patterns in IPF. IPF-related genes were identified based on differential expression analysis, univariate Cox regression analysis, the “Scissor” program, and the “Findmarkers” program. A combination of machine learning was employed to develop stable predictive and diagnostic signatures associated with IPF, based on the filtered relevant genes.

**Results:**

The stable PCDI.prog signature was established through the integration of 101 distinct machine-learning techniques, which exhibited superior efficacy in predicting outcomes in IPF patients through the validation of multiple datasets. Integrating PCDI.prog signature with patient clinical information, such as age, gender, and GAP score, enables the prediction of disease progression rates and patient survival. Additional PCDI.diag signature can offer insights into the early diagnosis of IPF.

**Conclusion:**

In summary, PCDI.prog signature and PCDI.diag signature offer critical insights for the early diagnosis, prognostic evaluation, and personalized treatment of IPF.

## 1 Introduction

Idiopathic pulmonary fibrosis (IPF) is a chronic interstitial lung disease characterized by progressive and irreversible fibrosis of the lung parenchyma (1). This fibrosis leads to a gradual decline in lung function. IPF patients have a bleak outlook, with a median survival of approximately 2–3 years from diagnosis (2) and a 5-year survival rate of less than 40% (3). Therefore, the early diagnosis and prognosis evaluation of IPF patients are very important. Cell death patterns can be categorized into two groups based on the rate of occurrence and susceptibility to medications or genes: accidental cell death (ACD) and programmed cell death (PCD) (4). ACD is an instantaneous and uncontrollable cell death caused by extreme biological, physical, chemical, or mechanical damage to the cytoplasmic membrane in the external environment, mostly manifested as necrosis (5). Previously, Ellson et al. (6) studied the significance of dangerous associated molecular patterns (DAMPs) in IPF in persistent inflammation and fibrosis, linking necrosis to the ongoing pathological processes in IPF. Furthermore, Emura et al. (7) studied the systemic consequences of acute exacerbation of IPF. They found that TNF-*α* positive cells were involved in the systemic circulation during acute exacerbation, which indicated that necrosis and inflammatory responses may extend beyond the lungs, leading to multi-organ damage and highlighting the systemic nature of the disease.

The pivotal role of PCD in maintaining *in vivo* homeostasis, host defense against pathogens, cancer development, and various other pathologies has been extensively documented (8). As research into cell death (CD) continues to deepen, numerous types of CD have been identified. However, the classification of certain types remains controversial. For example, autophagy, entosis, and methuosis continue to be subjects of debate. Some studies propose that these processes should be classified as PCD-vacuole presenting (9). Additionally, NETosis and netotic cell death were initially considered to contribute to immune responses by defending against pathogens. However, recent evidence suggests that they represent controlled forms of CD, potentially categorizing them as specialized types of PCD (10, 11). In this study, 20 distinct PCD patterns (Supplementary Table 1) were included.

PCD plays a critical role in the pathogenesis of IPF. Tsuburai et al. (12) demonstrated that adenovirus-mediated transfer and overexpression of heme oxygenase 1 (HO-1) cDNA in the lung can prevent bleomycin-induced pulmonary fibrosis by attenuating apoptotic cell death. This suggests that using HO-1 overexpression strategies could be effective in treating IPF. Carnesecchi et al. (13) further supported this by showing strong expression of NOX4, a key player in epithelial cell death, in the lungs of IPF patients. The interactions between epithelial cells and fibroblasts are also crucial in the development of pulmonary fibrosis. Sakai et al. (14) highlighted various signaling molecules involved in these interactions, including transforming growth factor-*β* and reactive oxygen species. Additionally, Mccubbrey et al. (15) found that deletion of the antiapoptotic protein c-FLIP from CD11bhi macrophages prevented the development of bleomycin-induced lung fibrosis, indicating the importance of cell death regulation in fibrotic processes. Ryter et al. (16) also highlighted the role of mitochondrial dysfunction in chronic lung diseases, including IPF, and its implications in regulating cell death programs like necroptosis. Baek et al. demonstrated that Spermidine mitigates the development of lung fibrosis caused by bleomycin by promoting autophagy and suppressing cell death generated by endoplasmic reticulum stress (ERS) in mice (17). Additionally, the suppression of ferroptosis and iron accumulation alleviated pulmonary fibrosis (18). Senescence plays a pivotal role in fibrosis, as fibroblasts transition into a senescent state and exhibit resistance to apoptosis (19). According to Hohmann et al. (20), quercetin has been shown to restore the susceptibility of senescent IPF fibroblasts to apoptotic stimuli, thereby alleviating bleomycin-induced pulmonary fibrosis. Quercetin demonstrates therapeutic potential by upregulating the expression of FasL receptor and caveolin-1, inhibiting AKT activation, and mitigating pulmonary fibrosis progression in aging mice. Additionally, Shen et al. revealed that senescent myofibroblasts resist apoptosis through the upregulation of BAX and the modulation of BCL-2/BCL-XL proteins, leading to BAX inactivation. The BAX activator BTSA1 promotes apoptosis in senescent cells and decelerates pulmonary fibrosis progression, offering a novel senescence clearance strategy for treating pulmonary fibrosis via the promotion of apoptosis in senescent cells (21). In conclusion, the studies suggest that PCD mechanisms play a critical role in the development of IPF.

**Figure 1:**
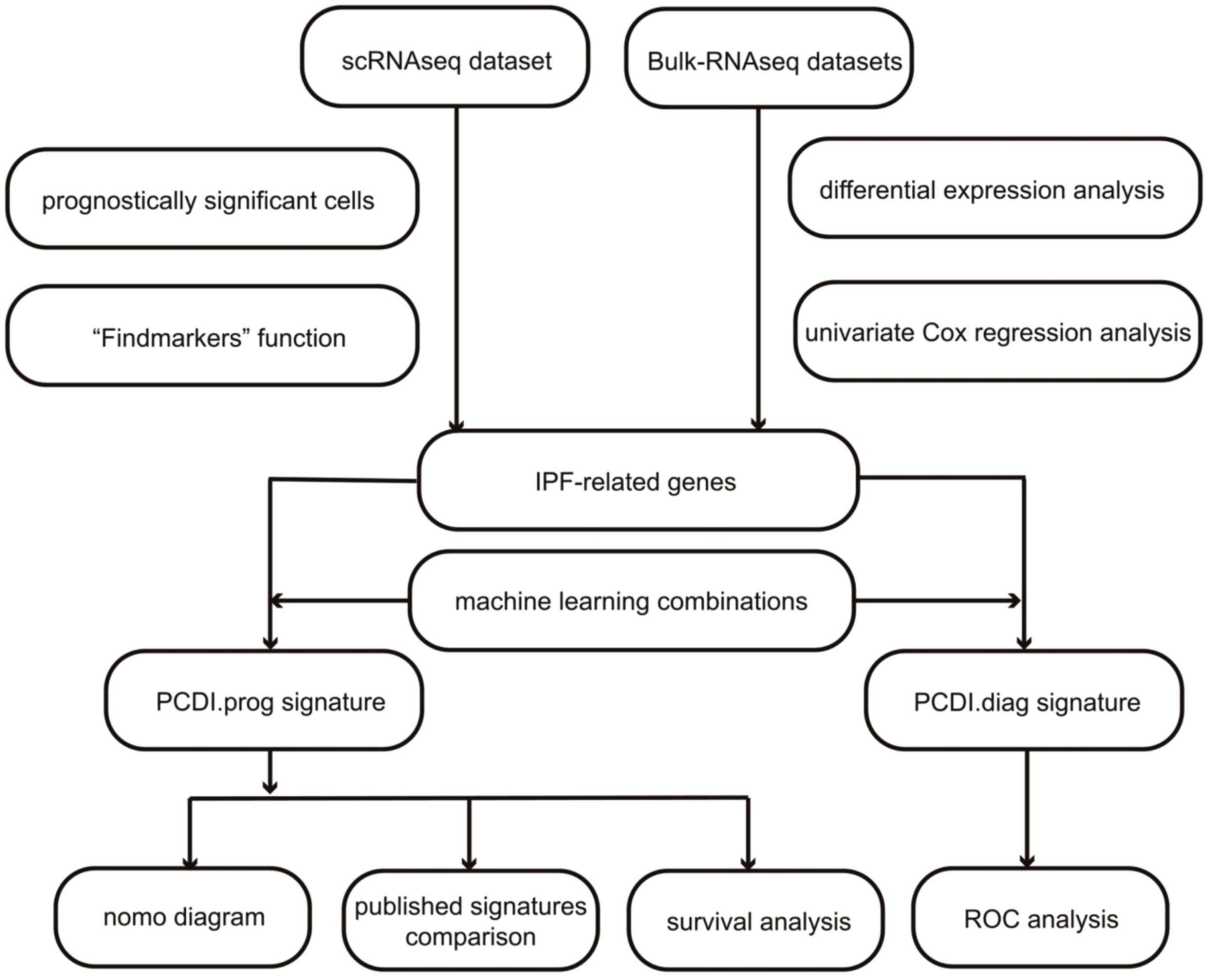
The study’s flowchart diagram.

Therefore, understanding the regulation of cell death pathways and targeting key molecules involved could provide potential therapeutic strategies for treating IPF. The study utilized microarray-based transcriptome profiling and single-nucleus RNA sequencing to identify and analysis of diverse PCD patterns in IPF. Prognostic genes were screened utilizing differential expression analysis and univariate Cox regression analysis based on the bulk RNAseq dataset. The “Scissor” R package was utilized to discern prognostically significant cells, utilizing the scRNA-seq dataset, and the “Findmarkers” function was employed to ascertain marker genes for prognostically significant cells based on the scRNA-seq dataset. IPF-related genes were identified by overlapping prognostic genes and marker genes. Based on the identified IPF-related genes, 101 distinct combinations of machine-learning techniques were employed to develop stable prognostic signatures. Validation of several datasets and feature comparison were employed to evaluate the superiority and generalizability of prognostic signature. Additionally, the study employed a combination of machine learning techniques to develop a signature for diagnosing IPF, which may serve as a reference for the early identification of IPF. Figure 1 illustrates the precise procedure of the study.

## 2 Materials and methods

### 2.1 The availability of PCD-related genes

Key regulatory genes associated with 20 PCD patterns were sourced from multiple repositories, including the Kyoto Encyclopedia of Genes and Genomes (KEGG) Database (22), the GeneCards Database (23), the Molecular Signatures Database (24), the Reactome Database, FerrDb database, the Human Autophagy Database, and published articles. The comprehensive gene list comprising 20 distinct PCD patterns (Supplementary Table 2), encompassing 7 alkalosis-related genes (25), 338 anoikis-related genes (26, 27), 579 apoptosis-related genes, 19 Cuproptosis-related genes (28–30), 10 disulfidptosis-related genes (31), 23 entosis-related genes, 15 entotic cell death-related genes, 34 immune cell death (32), 220 lysosome dependent cell death-related genes, 8 methuosis-related genes, 39 MPT driven necrosis, 101 necroptosis-related genes, 24 NETosis-related genes, 8 netotic cell death-related genes, 5 oxeiptosis-related genes, 9 parthanatos-related genes, 59 parapoptosis-related genes (33), 52 pyroptosis-related genes (34), 367 autophagy-related genes and 88 ferroptosis-related genes.

### 2.2 Differential expression analysis

The R package “limma” was employed to extract differentially expressed genes (DEGs) between the IPF cohort and health control cohort in the IPF dataset (*p* value < 0.05).

### 2.3 Functional enrichment analysis

Further, the study performed the gene ontology (GO), and Kyoto encyclopedia of genes and genomes (KEGG) enrichment analysis of all the DEGs using the “ClusterProfiler” R package.

### 2.4 Single-cell analysis

The GSE122960 (35) dataset provided scRNA-seq information from the lung tissue of three IPF patients and three healthy donors. To ensure the correctness and reliability of the information, the scRNA-seq information underwent stringent screening and quality control. It excluded cells with mitochondrial gene expression levels beyond 20% from the dataset. Furthermore, cells containing fewer than 300 genes and genes represented by fewer than five cells are excluded. The selected cells were subsequently subjected to downstream analysis. The scRNA-seq information was normalized by the “NormalizeData” function, which was then converted to Seurat objects and the first 1,500 highly variable genes were identified using the “FindVariableFeatures” function. Afterward, the “RunPCA” function of the “Seurat” R package was applied to perform principal component analysis (PCA) to reduce the dimensionality of the scRNA-seq data based on the top 1,500 genes. The functions “FindNeighbors” and “FindClusters” were used for cell clustering analysis.

Cell clusters were annotated using reference data from the Human Cell Atlas and were subsequently refined based on specific cell biomarkers including Type I Alveolar Epithelial (AT1) Cells (AGER and RTKN2), Type II Alveolar Epithelial (AT2) Cells (LAMP3), Club Cells (SCGB3A2), Ciliated Cells (TPPP3), Basal Cells (KRT5 and TP63), Macro/Mono (CD68 and CD14), Dendritic Cells (CLEC10A), T/NKT Cells (CD3D), Plasma Cells (IGHG4), B Cells (MS4A1), Mast Cells (TPSB2), Endothelial Cells (VWF), Fibroblasts (DCN).

Furthermore, the “Scissor” R package was employed to integrate scRNA-seq information, bulk RNA-seq information, and phenotypic information to identify cell subpopulations that display significant correlations with the prognosis of IPF patients. The “IPF-related genesEA” R package was employed to conduct gene set enrichment analysis (GSEA) on the scRNAseq dataset based on the “AUCell,” “UCell,” “singscore,” and “ssgsea” algorithms.

### 2.5 Construction and validation of prognostic signature

The datasets enrolled in the study were obtained from the Gene Expression Omnibus (GEO) database.^1^ The prognosis signature was constructed based on four datasets, including GSE27957 (36), GSE28042 (36), GSE70866 (37), and GSE93606 (38). GSE70866 was used as the training set with various clinical features and samples, while the other three data sets were used as the validation sets. Each gene expression was transformed into a z-score across patients in all cohorts. The screening criteria for samples were as follows: (1) The individual received a diagnosis of IPF; (2) The IPF patient possessed comprehensive bulk-RNAseq information; (3) The IPF patient had a survival information record (refer to Supplementary Table 3 for detailed clinical parameters).

To enhance the precision and consistency of the prognostic signature, the study incorporated ten machine-learning algorithms into the analysis. These algorithms encompass random survival forest (RSF) (39), elastic network (Enet), Lasso, Ridge, Stepwise Cox, CoxBoost (40), partial least squares regression for Cox (plsRcox) (41), supervised principal components (SuperPC) (42), generalized boosted regression modeling (GBM) (43), and survival support vector machine (survival-SVM) (44) (refer to Supplementary Table 4 for details). Several algorithms have demonstrated the ability to perform feature selection, which was employed to screen crucial genes, including Lasso, stepwise Cox, Coxboost, and RSF algorithms. Ultimately, we developed 101 algorithmic combinations to enhance the identification of prognostic signatures with commendable accuracy and stability. Subsequent survival analysis and signature comparison were employed to assess the superiority and generalizability of the prognostic signature.

### 2.6 Construction and validation of the diagnostic signature

GSE150910 (45), GSE24206 (46), GSE28042 (36), GSE53845 (47), and GSE70866 (37) from the GEO database were enrolled to develop the diagnostic signature. The GSE150910 dataset served as the training cohort, while four datasets were utilized as the validation cohorts.

An efficient diagnostic signature for accurate prediction of IPF was developed by merging diverse machine-learning techniques based on PCD-related genes. The machine learning algorithms encompass Lasso, Ridge, Stepglm, XGBoost, Random Forest (RF) (39), Elastic Net (Enet), Partial Least Squares Regression for Generalized Linear Models (plsRglm), Generalized Boosted Regression Modeling (GBM), Naive Bayes (48), Linear Discriminant Analysis (LDA) (49), Generalized Linear Model Boosting (glmBoost), and Support Vector Machine (SVM). Ultimately, a total of 101 signatures were developed. For each signature, the area under the receiver operating characteristic curve (AUC) values were calculated across all validation datasets, and the signature exhibiting the highest average AUC in the validation cohort was deemed the superior diagnostic signature, owing to overfitting in the training cohort. The diagnostic model’s superiority was established by comparing its AUC value with that of the clinical features.

### 2.7 Drug sensitivity analysis

The “pRRophetic” (50) R package was applied to predict the therapeutic response of IPF patients to common drugs, and the value of the PCDI.prog signature in guiding the selection of drugs for IPF patients was assessed based on the IC50 values in different PCDI.prog score groupings.

### 2.8 Statistical analysis

Statistical differences between groups were determined by Student’s t-test for normally distributed variables, and for non-normally distributed variables, statistical differences between groups were determined by the Wilcoxon test. The statistical studies were conducted using the R project (version 4.3.3).

## 3 Results

### 3.1 The pathway activity profiling of PCD patterns in normal and fibrotic lung tissues

**Figure 2:**
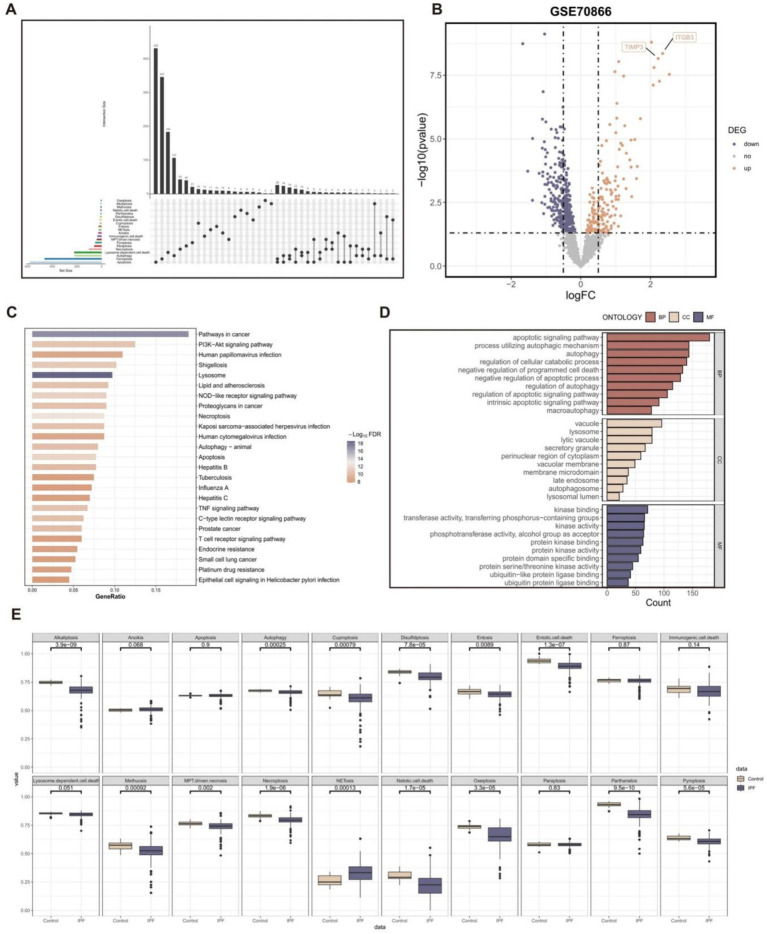
The pathway activity profiling of PCD patterns in normal and fibrotic lung tissues. **(A)** The Upset plot displaying diverse PCD patterns and key regulatory genes. **(B)** Volcano plot of the PCD-related DEGs. Points with labels are obvious DEGs with *p*-value < 0.05 **(C)** GO enrichment analyses based on the DEGs. **(D)** KEGG enrichment analyses based on the DEGs. **(E)** Box plot displaying the pathway activity profiling of PCD patterns in normal and fibrotic lung tissues based on the “ssGSEA” algorithm.

In this study, we collected a total of 20 PCD patterns and 2013 key regulatory genes from the existing published articles and online databases. We removed 449 duplicate gene symbols, resulting in 1,564 PCD-related genes for subsequent analysis (Figure 2A).

A total of 524 genes with significant differential expression (*p*-value < 0.05) were identified in the GSE70866 cohort (Figure 2B). Furthermore, the DEGs are associated with many PCD pathways and signal transduction pathways related to IPF, as demonstrated by the KEGG and GO enrichment studies (Figures 2C,D).

In addition, the study investigated the profiling of pathway activity in normal and fibrotic lung tissues to analyze PCD patterns (Figure 2E). The results suggested that except NETosis pattern, the activity of other PCD patterns decreased in fibrotic lung tissue. The vast majority of PCD patterns showed significant differences in activity in normal and fibrotic lung tissues.

### 3.2 Dissection of the microenvironment of IPF based on PCD patterns

**Figure 3:**
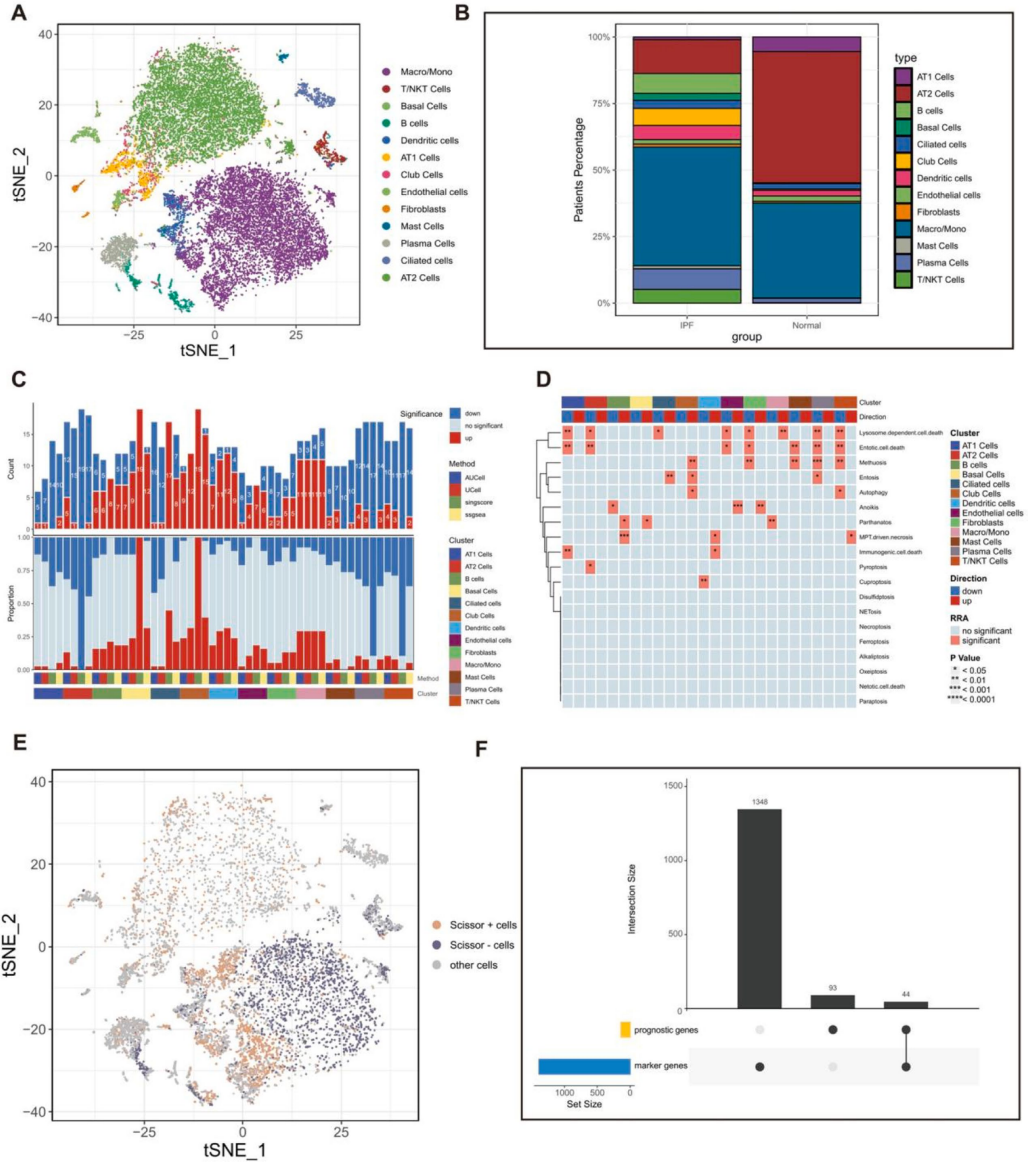
The scRNAseq analysis of fibrotic lung tissues. **(A)** The t-SNE plot displaying the composition of cells in the microenvironment of IPF. **(B)** Bar plot displaying the proportion of each identified cell in IPF and normal samples. **(C)** Histogram displaying the number of PCD patterns in different cell types. **(D)** The heatmap displaying the PCD patterns in different cell types. **(E)** The t-SNE plot displaying the distribution of prognostically significant cells in the microenvironment of IPF. **(F)** Upset plot displaying the 44 IPF-related genes, which were derived from the intersection of 137 prognostic genes obtained from bulk RNAseq dataset and 1,392 marker genes of prognostically significant cells. Scissor + cells, Cells positively associated with OS in patients with IPF; Scissor − cells, Cells negatively associated with OS in patients with IPF; other cells, Cells unrelated to OS in patients with IPF.

A total of 9,295 cells, obtained from lung tissue samples of three IPF individuals and three healthy individuals, were grouped into 23 unique clusters using the “Seurat” and “clustree” R packages (Supplementary Figures 1A,B). A total of 13 unique cell types were distinguished using particular cell marker genes (Supplementary Figure 1C; Figure 3A). The population of epithelial cells in the lungs of individuals with IPF was shown to have a higher percentage of airway epithelial cells (specifically basal cells, ciliated cells, and club cells) and a notable drop in alveolar epithelial cells (AT1 cells and AT2 cells). This pattern aligns with prior findings (51, 52) (Figure 3B).

Furthermore, the study provided an initial description of the variations in PCD-related pathway activity among different cell subtypes in fibrotic lung tissues based on the integration of four algorithms (Figures 3C,D). As we all know, fibroblasts play a crucial role in the development of IPF by promoting the production of collagen and cell proliferation, leading to the formation of fibrous tissue (53). The study suggested that there is a difference in the behavior of PCD patterns in fibroblasts in fibrotic lung tissues, especially heightened activity in the anoikis pathway in fibroblasts. Previous studies have demonstrated that anoikis takes place in lung fibroblasts during the development of fibrosis and has been identified as a significant factor in the progression of IPF (54), indicating possible treatment targets for IPF.

### 3.3 Identification of IPF-related genes

A univariate regression analysis was conducted on a set of 524 DEGs to find 137 prognostic genes. Furthermore, the “Scissor” R package was employed to integrate the scRNA-seq GSE122960 dataset and the bulk-RNAseq GSE70866 dataset. We identified 1,934 cells positively associated with OS in patients with IPF and 2,041 cells that were negatively associated with OS in patients with IPF (Figure 3E). Additionally, the “Findmarkers” function was used to identify 1,392 marker genes for cells that had prognostic significance. Ultimately, a total of 44 IPF-related genes (Supplementary Table 5) were determined by identifying the common genes that serve as both prognostic genes and marker genes (Figure 3F).

### 3.4 Construction of the PCDI.prog signature

Subsequently, we employed a total of 10 distinct machine-learning algorithms to develop and construct 101 prognostic signatures. The robustness of these signatures was evaluated using a 10-fold cross-validation approach in four distinct cohorts (GSE70866 as the training cohort, and three external validation cohorts including GSE27957, GSE28042, and GSE93606 cohort).

**Figure 4:**
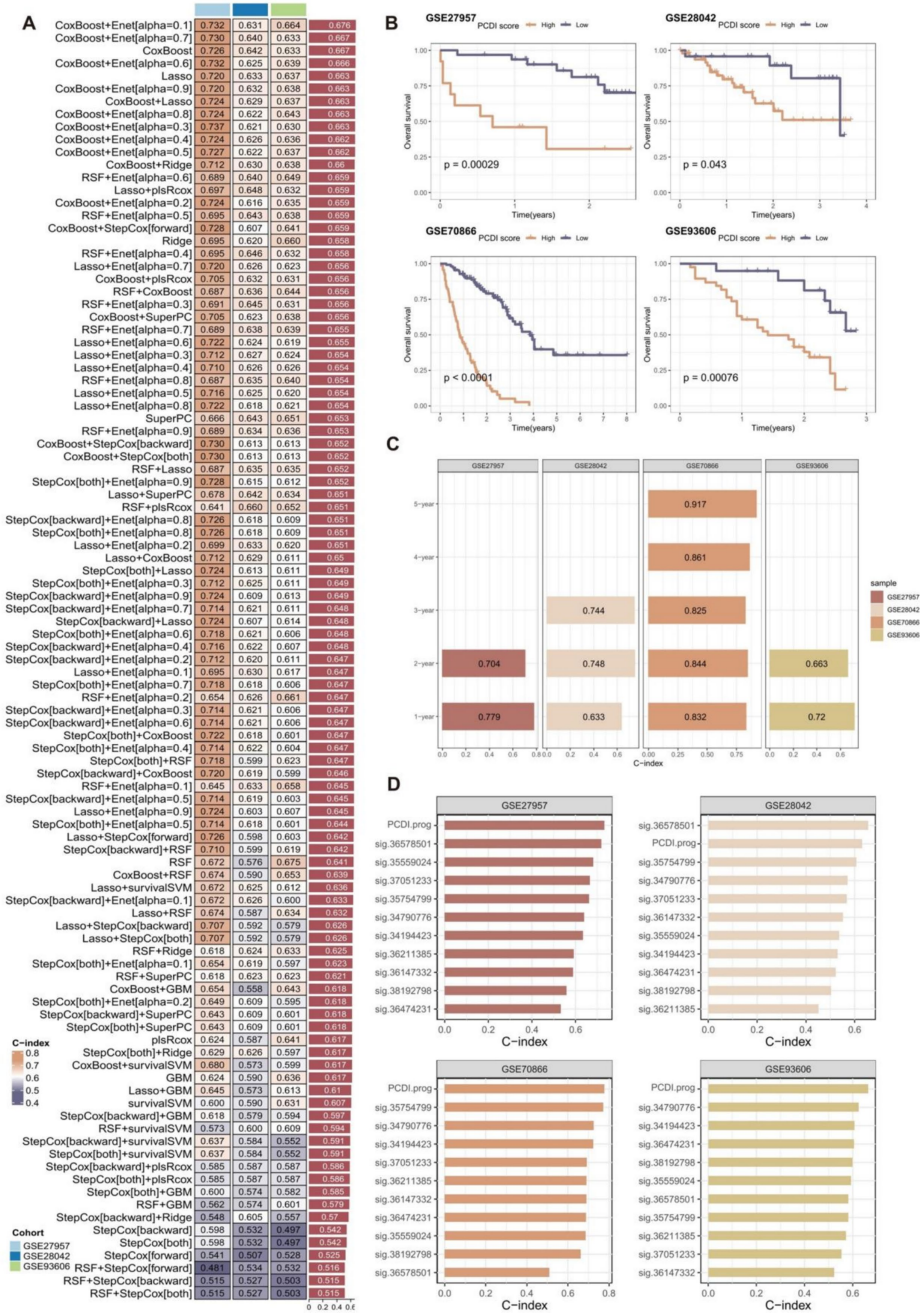
Construction and validation of the PCDI.prog signature. **(A)** A total of 101 combinations of machine learning algorithms for the PCDI.prog signatures via a 10-fold cross-validation framework based on the GSE70866 cohort. The C-index of each model was calculated across validation datasets, including the GSE27957, GSE28042, and GSE93606 cohort. **(B)** Kaplan–Meier survival curve of OS between patients with high-PCDI.prog scores and low-PCDI.prog scores in the training and validation cohorts. **(C)** ROC analysis of PCDI.prog signature in the training and validation cohorts. **(D)** C-index comparison of PCDI.prog signature and 10 previously published signatures in the training and validation cohorts.

The best-performing predictive signature was determined as the signature with the greatest mean C-index in three external validation cohorts, due to overfitting in the training cohort (Figure 4A). The findings indicated that the CoxBoost + Enet[alpha = 0.1] algorithm combination demonstrated the highest average C-index (0.676), making it the optimal algorithm combination for developing the programmed cell death index prognostic (PCDI.prog) signature.

Afterward, the PCDI.prog score for each patient was calculated. The samples were then categorized into the low PCDI.prog score group and the high-PCDI.prog score group based on the optimal threshold value of the PCDI.prog score determined using the R package “survminer.” Subsequently, KM survival analysis and assessment of prognostic performance were conducted. Demonstrating a substantial difference in OS between the low-PCDI.prog score group and the high-PCDI.prog score group in all four cohorts (Figure 4B).

### 3.5 Validation and clinical application of PCDI.prog signature

The accuracy and reliability of the PCDI.prog signature predicting 1-,2-, 3-, 4-, and 5-year survival of IPF patients was supported by empirical evidence that the area under the curve (AUC) values exceeded 0.65 in multiple distinct cohorts (Figure 4C).

Furthermore, due to the scarcity of prognostic models for non-tumor diseases compared to tumors, and the limited availability of datasets including comprehensive gene expression data for genes associated with signatures, a total of 10 prognostic models published in IPF were ultimately gathered from the existing literature. The features encompass a range of biological processes observed in the IPF cohort, such as hypoxia, autophagy, pyroptosis, epithelial-mesenchymal transition, epigenetic regulation, and inflammation (Supplementary Table 6). These features were then compared to the C-index of the PCDI.prog signature. The findings indicated that the PCDI.prog signature exhibited superior performance compared to the majority of the signatures within their respective categories (Figure 4D).

**Figure 5:**
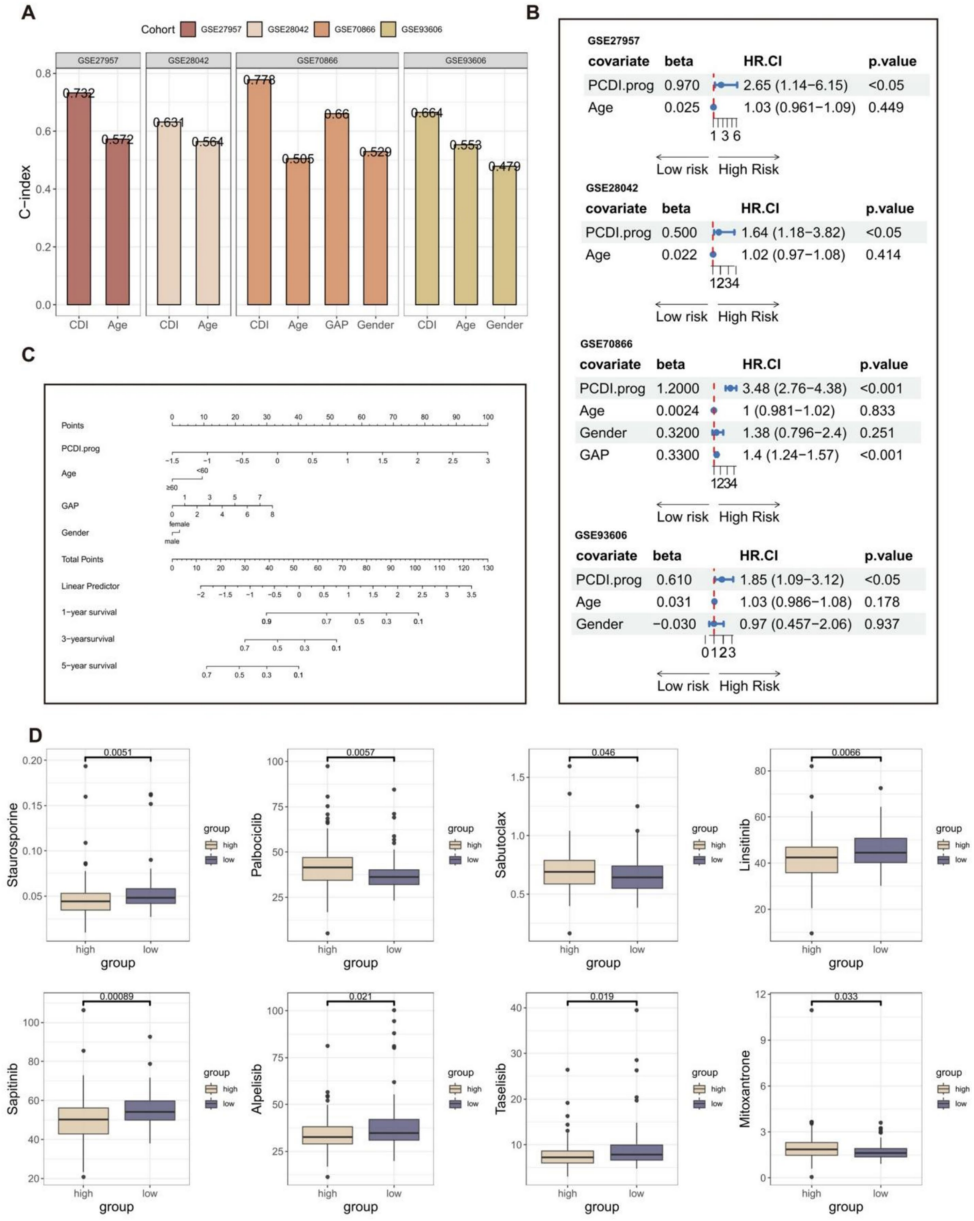
Establishment of the nomogram survival model. **(A)** The predictive performance of the PCDI.prog signature was compared with common clinical variables in the training and validation cohorts. **(B)** Univariate analysis for the clinicopathologic characteristics and PCDI.prog in the training and validation cohorts. **(C)** A nomogram was established to predict the prognostic of IPF patients based on PCDI.prog score, age, gender, and GAP score. **(D)** Drug sensitivity analysis of IPF patients in different PCDI.prog score group.

In addition, we compared the predictive value of the PCDI.prog signature with other clinical variables (Figure 5A). The C-index of the PCDI.prog signature was significantly higher than other clinical variables, covering GAP score, age, and gender. The univariate Cox regression analysis showed that compared with other features, PCDI.prog signature was regarded as a risk factor (Figure 5B). Finally, to facilitate clinical application, a nomogram was created, integrating the factors of age, gender, GAP score, and PCDI.prog score (Figure 5C).

The “pRRophetic” R package is a computational model that predicts chemotherapy responses based on gene expression data (55). Nintedanib, an orally administered small molecule tyrosine kinase inhibitor initially designed for lung cancer, has been approved for the treatment of IPF. It serves as an example of an anti-tumor medication associated with medications used for IPF. Furthermore, the addition of pirfenidone (56) can diminish the efficacy of the paclitaxel and carboplatin combination. Therefore, we believe that the identification of related anti-tumor drugs has potential significance for IPF treatment. Eight potential drugs for IPF patients were obtained through drug susceptibility analysis (Figure 5D). Individuals with low PCDI.prog scores exhibited a notable rise in sensitivity to staurosporine, linsitinib, sapitinib, taselisib, and alpelisib. Individuals with high PCDI.prog scores exhibited a notable rise in sensitivity to palbociclib, sabutoclax, and mitoxantrone. It is suggested that the PCDI.prog signature has a potential guiding effect on the treatment of IPF patients.

### 3.6 Construction and validation of PCDI.diag signature

**Figure 6:**
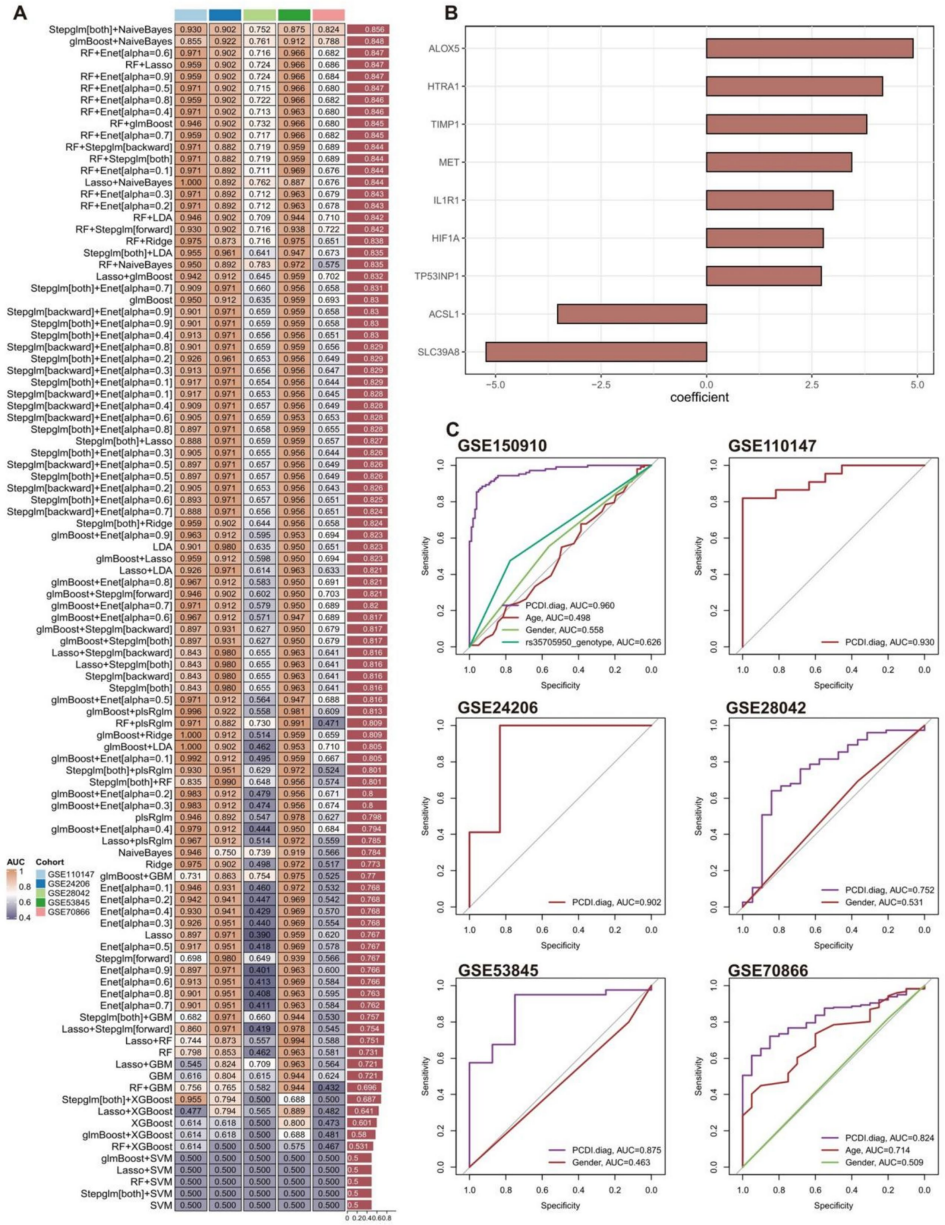
Construction and validation of PCDI.diag signature. **(A)** A total of 101 diagnostic signatures were assessed utilizing a 10-fold cross-validation framework, and the AUC value for each signature was subsequently computed across all validation cohorts. **(B)** Nine crucial genes identified by the Stepglm[both] algorithm. **(C)** ROC curves illustrate the efficacy of IPF predictions made by PCDI.diag signature, age, rs35705950 genotype, and gender in the training and validation cohorts.

A total of 101 diagnostic signatures of GSE150910 were evaluated using 10-fold cross-validation (Figure 6A). The AUC values for each signature were computed across all validation datasets, including GSE110147, GSE24206, GSE28042, GSE53845, and GSE70866. The combination of Stepglm[both] and NaiveBayes was recognized as the optimal signature, yielding the greatest average AUC value of 0.856. The Stepglm[both] algorithm identified 9 crucial genes (SLC39A8, HIF1A, TIMP1, ACSL1, ALOX5, MET, IL1R1, HTRA1, TP53INP1) (Figure 6B), whereas the NaiveBayes algorithm developed the programmed cell death diagnostic (PCDI.diag) signature.

The diagnostic effectiveness of the PCDI.diag signature in predicting IPF was compared with other clinical factors in both the training and validation cohorts (Figure 6C). Regrettably, the GSE110147 and GSE24206 cohorts lack comprehensive clinical data. The findings indicated that the PCDI.diag signature exhibited markedly superior accuracy compared to other clinical features in GSE150910, GSE28042, GSE53845, and GSE70866, including age, rs35705950_genotype, and gender. The results suggest that the PCDI.diag signature, derived from nine crucial genes, may offer novel insights into the preliminary diagnosis of IPF. It is worth noting that the AUC values of gender in multiple datasets are weak, which means that gender cannot well distinguish the IPF patient group from the healthy control group. Previous studies have also shown that Although IPF seems to affect older men predominantly, the true prevalence of IPF in women is difficult to establish, and women may be underdiagnosed while men are overdiagnosed with IPF based on gender alone (57).

## 4 Discussion

IPF is a lethal condition characterized by the progressive fibrosis of lung tissue, defined by the aberrant proliferation of interstitial lung cells, an inflammatory response, the advancement of fibrosis, and a gradual decline in pulmonary function. The precise pathophysiology of IPF remains incompletely elucidated, and the current study indicates a significant association between PCD and IPF. The cytokines and necrotic materials released during PCD not only directly promote the proliferation and activation of fibroblasts but also exacerbate the local inflammatory response by attracting immune cells, such as macrophages and neutrophils, ultimately resulting in the worsening of pulmonary fibrosis. The regulatory genes of PCD typically participate in this process; therefore, analyzing the expression patterns of these genes can yield critical insights for the early diagnosis, prognosis evaluation, and personalized treatment of IPF.

The study utilized microarray-based transcriptome profiling and single-nucleus RNA sequencing to identify and analysis of diverse PCD patterns in IPF. A subsequent study identified IPF-related genes that are crucial in the advancement of IPF. The stable PCDI.prog signature was established through the integration of 101 distinct machine-learning techniques, which exhibited superior efficacy in predicting outcomes in IPF patients through the validation of multiple datasets. Integrating PCDI.prog signature with patient clinical information, such as age, gender, and GAP score, enables the prediction of disease progression rates and patient survival. Additional PCDI.diag signature can offer insights for the early diagnosis of IPF. To facilitate clinical application, PCDI. Prog signature and PCDI. Diag signatures are integrated into the “PCDI” R package and are available at https://github.com//sjz17//IPF.

Furthermore, the majority of genes enrolled in the PCDI.prog signature and PCDI.diag signature have been confirmed to be involved in PCD patterns and regulating fibrotic processes (Supplementary Table 7). Interestingly, TIMP1, MET, and HTRA1 are present in both the PCDI.prog and PCDI.diag signatures.

The studies from Jamie et al. (58) showed that TIMP1 may play a role in regulating fibrosis in the microenvironment of IPF and fibroblasts are the most important cells in driving TIMP-1 dysregulation. Shibnath et al. (59) have shown an increased MET expression in lung fibroblasts from patients with pulmonary fibrosis as compared with lung fibroblasts from normal people. Moreover, MET has been implicated in driving profibrotic phenotypes and leading to pulmonary fibrosis (60). In the realm of pulmonary pathology, quantitative proteomic analysis has recognized HTRA1 as a protein implicated in tissue remodeling associated with IPF (61). Additionally, the overexpression of HTRA1 in BAL cells from patients with IPF was associated with a significantly poor prognosis (62). Furthermore, Chio et al. (63) demonstrated that the loss of function of HtrA1 has been shown to induce EMT by activating TGF-b and Notch signaling pathways, contributing to the progression of fibrosis in the lungs (64).

Despite the significant promise of prognostic and diagnostic signatures derived from PCD-related genes, several problems persist. Initially, IPF patients exhibit significant heterogeneity at both the molecular level and in clinical manifestations, leading to considerable variability in the expression of certain genes among people, which impacts the model’s accuracy. Secondly, IPF is an uncommon condition, particularly in its initial phases, and the patient cohort is quite small, potentially impacting the precision of genetic screening and the robustness of the model. Ultimately, PCD constitutes only a component of IPF progression, and models must consider environmental factors (such as smoking, genetic predisposition, immunological responses, and additional variables). Future research can enhance the diagnostic and prognostic signatures of IPF through extensive, multicenter clinical trials, liquid biopsy methodologies, and multidimensional information integration to offer more accurate and personalized therapy alternatives for clinical practice.

## 5 Conclusion

In summary, the PCDI.prog signature and PCDI.diag signature offer critical insights for the early diagnosis, prognostic evaluation, and personalized treatment of IPF.

## Data Availability

The original contributions presented in the study are included in the article/Supplementary material, further inquiries can be directed to the corresponding authors.

## Footnotes

^1^ http://www.ncbi.nlm.nih.gov/geo/
