# Supplementary material for: Identification and analysis of diverse programmed cell death patterns in idiopathic pulmonary fibrosis using microarray-based transcriptome profiling and single-nucleus RNA sequencing
Source: Front Med (Lausanne). 2025 Jun 18;12:1534903. doi: 10.3389/fmed.2025.1534903 (PMC12216088; doi:10.3389/fmed.2025.1534903)
Supplement: Supplementary file 1 [file Data_Sheet_1.pdf]

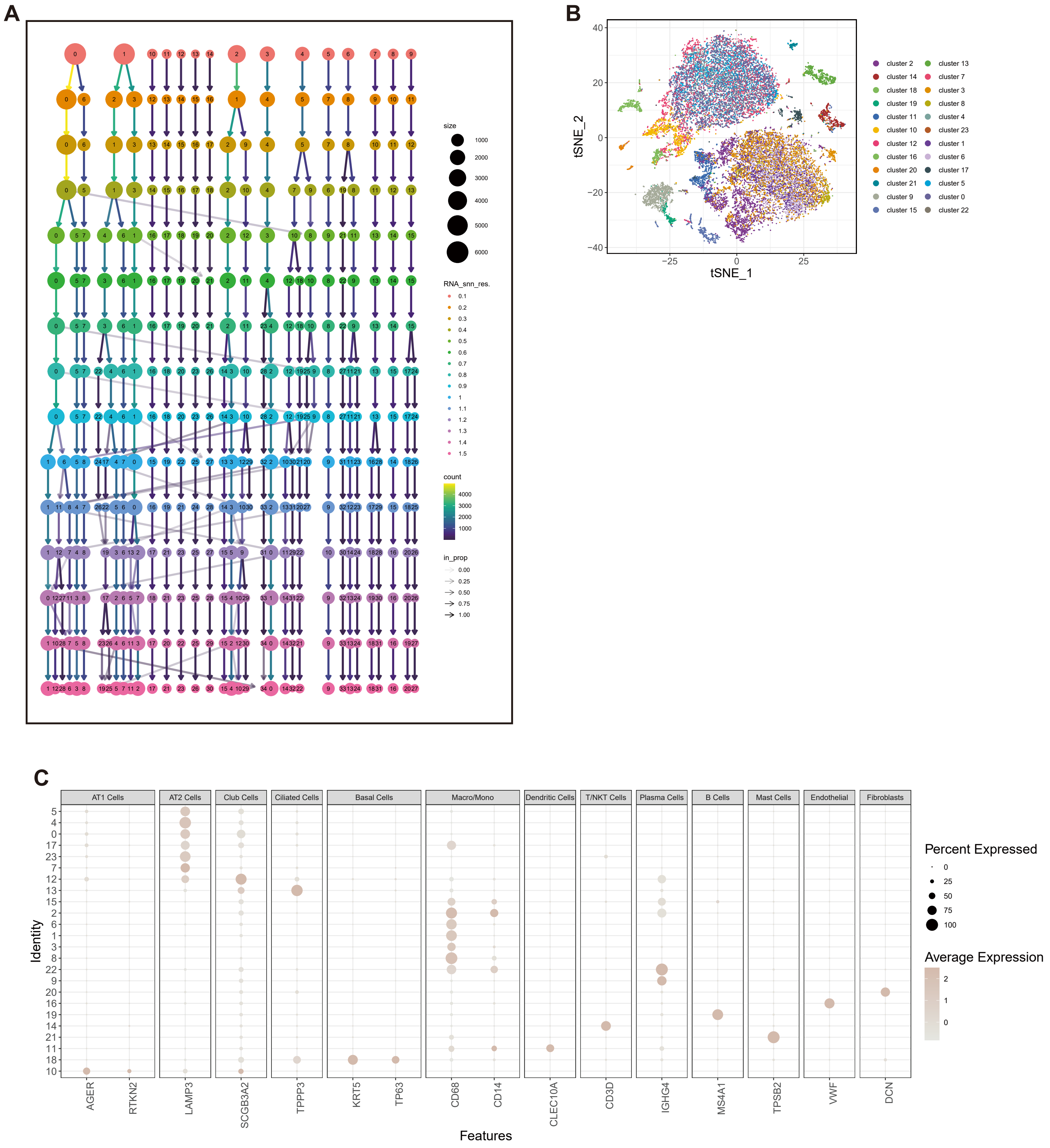

SFigure 1 Annotation of scRNA-seq data

(**A**) The clustering tree displaying total scRNA-seq data analyzed at different resolutions (**B**)The t-SNE plot displaying the cell clusters in the microenvironment of IPF. (**C**) Dot plot displaying expression of cell-type markers across cell clusters. Dot size indicates the percentage of expressed cells, colored by their relative expression levels
